# The Impact of Common Epidemiological Factors on Gray and White Matter Volumes in Magnetic Resonance Imaging–Is Prevention of Brain Degeneration Possible?

**DOI:** 10.3389/fneur.2021.633619

**Published:** 2021-07-13

**Authors:** Jagoda Jacków-Nowicka, Przemysław Podgórski, Joanna Bladowska, Dorota Szcześniak, Joanna Rymaszewska, Katarzyna Zatońska, Katarzyna Połtyn-Zaradna, Andrzej Szuba, Marek Sa̧siadek, Anna Zimny

**Affiliations:** ^1^Department of General and Interventional Radiology and Neuroradiology, Wroclaw Medical University, Wrocław, Poland; ^2^Department of Psychiatry, Wroclaw Medical University, Wrocław, Poland; ^3^Department of Social Medicine, Wroclaw Medical University, Wrocław, Poland; ^4^Department of Angiology, Hypertension and Diabetology, Wroclaw Medical University, Wrocław, Poland

**Keywords:** brain aging, brain volumetry, white matter hyperintensities, epidemiological factors, prevention of brain degeneration

## Abstract

**Introduction:** The aim of the study was to evaluate the impact of multiple risk factors (age, diabetes, hypertension, hyperlipidemia, BMI, smoking, alcohol) on the gray and white matter volumes as well as on the burden of white matter hyperintensities (WMH).

**Material and Methods:** The study group consisted of 554 subjects (age range: 50–69 yrs, F/M: 367/187) recruited from the larger cohort of the Polish fraction of the Prospective Urban Rural Epidemiological (PURE) study. The participants answered questionnaires about their lifestyle, underwent physical and psychological examination (MoCA test), laboratory blood tests followed by brain MRI. Volumetric measurements of the total gray matter (GMvol), total white matter (WMvol) and WHM (WMHvol) normalized to the total intracranial volume were performed using the Computational Anatomy Toolbox 12 (CAT12) and Statistical Parametric Maps 12 (SPM12) based on 3D T1-weighted sequence. The influence of risk factors was assessed using multiple regression analysis before and after correction for multiple comparisons.

**Results:** Older age was associated with lower GMvol and WMvol, and higher WMHvol (*p* < 0.001). Smaller GMvol volume was associated with higher WMHvol (*p* < 0.001). Higher WMHvol was associated with hypertension (*p* = 0.01) and less significantly with hyperlipidemia (only before correction *p* = 0.03). Diabetes, abnormal BMI, smoking and alcohol intake did not have any significant impact on GMvol, WMvol or WMHvol (*p* > 0.05). MoCA score was not influenced by any of the factors.

**Conclusions:** Gray matter loss is strongly associated with the accumulation of WMH which seems to be potentially preventable by maintaining normal blood pressure and cholesterol levels.

## Introduction

Brain aging is a complex process affecting both gray and white matter. It is a physiological process that begins in the fourth and peaks in the seventh decade of life (1, 2). Physiological degeneration of the gray and white matters is the result of alterations in their microstructure and biochemistry leading not only to the loss of brain cells but also to the accumulation of iron, amyloid plaques, neurofibrils, lipofuscin and neuromelanin, as well as a decrease in the number of neurotransmitters such as dopamine and serotonin (3, 4). At a molecular level there are also some other factors that can affect the process of brain aging such as dysregulation of calcium metabolism, abnormalities in glucose metabolism or mitochondrial dysfunction (5, 6).

Brain aging is influenced by many factors including genetic, environmental, or coexisting diseases. Cerebral vascular pathology strongly contributes to acceleration of brain degeneration, especially cerebral small vessel disease (CSVD) causing lacunar infarcts, microbleeds and white matter hyperintensities (WMH) leading to white matter damage (7). Recently, WMH have been of a great interest since they are associated with the increased risk of cognitive impairment and vascular dementia (8). Their etiology and pathophysiology are not yet fully understood, however hypoxic-ischemic axonal loss and demyelination, hypoperfusion due to altered cerebrovascular autoregulation as well as blood-brain barrier dysfunction have been established as leading pathophysiological causes (9). Histopathological findings in regions of WMH are myelin paleness, tissue rarefaction with loss of myelin and axons, as well as mild gliosis (10).

It has been shown that several hormones, other comorbidities as well as environmental and genetic factors may also have an impact on gray and white matter degeneration (11–13). Atrial fibrillation, genetic variants in the APOEe4gene, low mental activity and passive lifestyle may accelerate brain atrophy (9). Other studies revealed that hypertension, diabetes, hypercholesterolemia may also have a significant impact on this process while healthy lifestyle, balanced diet, regular physical activity, low or no alcohol consumption and prevention of cardiovascular diseases directly reduce the occurrence of WMH and the risk of white matter atrophy (4, 9, 14, 15).

Pathological acceleration of gray and white matter degeneration is closely linked to cognitive impairment and dementia. In the past decades dementia has become a huge clinical, social and economic problem reaching a status of pandemic affecting 50 million people worldwide with nearly 10 million new cases every year according to the WHO report from 2020. It has been reported that markers of CSVD are not only common in individuals diagnosed with vascular dementia but also in patients with Alzheimer's disease (AD) and, after accounting for non-independence between risk factors, about one-third of AD cases worldwide might be attributed to potentially modifiable factors (16, 17). It is also well-known that brain degeneration in AD and other dementias accumulates sub-clinically over years leading finally to irreversible severe brain damage.

The association of different risk factors with brain atrophy and WMH has already been reported in the literature however the results are often contradictory. Majority of the articles focus on evaluation of the influence of single factors while they usually do not occur isolated but as a combination of multiple risk factors (15, 18, 19).

The aim of this cross-sectional study was to evaluate the impact of multiple common demographic and epidemiological factors such as age, sex, diabetes, hypertension, hyperlipidemia, body weight, smoking and alcohol consumption on the volumes of the gray and white matter and on the presence of WMH in a large cohort of 554 community-based subjects recruited from the Polish sub-study of the large multinational Prospective Urban Rural Epidemiological (PURE) study. To our knowledge this is the first report showing the results of associations between multiple factors and brain volumetry as well as the presence of WMH from a large cohort of subjects representing Central Europe Caucasian population. Since there is no effective treatment available, knowledge of risk factors of lower brain volumes and white matter damage, which may be specific for population and geographic regions, seems to be of great importance in the successful prevention of cognitive impairment and dementia.

## Materials and Methods

The study group consisted of 554 subjects (367 women and 187 men) recruited from a larger cohort of 1,269 participants of the Polish sub-study of the multinational Prospective Urban Rural Epidemiological (PURE) study. All participants underwent demographic and risk factor assessment followed by psychological evaluation and magnetic resonance imaging (MRI) of the brain. The study exclusion criteria were: age below 50 and above 69 years, contraindications to MRI (including mainly a pacemaker and other contraindicated body implants, as well as severe claustrophobia), history of stroke or dementia as well as other significant neurological or psychiatric diseases.

An informed consent was obtained from all individual participants involved in the study. The study protocol was approved by the local Bioethical Committee (permission No. KB-32/2016).

### Demographic and Risk Factor Assessment

Demographic and medical information including: age, sex, diabetes, hypertension, hyperlipidemia, body max index (BMI), smoking status, alcohol consumption were collected using standardized PURE questionnaires followed by laboratory blood assessment and physical examination. Diabetes was defined as fasting blood glucose ≥126 mg/dl (7.0 mmol/l) or currently treated diabetes while hyperlipidemia as total cholesterol ≥200 mg/dl. Hypertension was defined as a systolic blood pressure (SBP) ≥135 mm Hg or diastolic blood pressure (DBP) ≥85 mmHg or currently treated hypertension. Body mass index was calculated as weight (kg) divided by height (m) squared. Abnormal weight was defined as BMI ≥25 and normal <25. Information on smoking status and alcohol consumption was collected as self-reported answers “yes” or “no” in the questionnaires. The smoking group included current and former smokers. Alcohol intake was negative when no alcohol consumption was reported.

### Cognitive Assessment

Cognitive assessment was performed using the Montreal Cognitive Assessment (MoCA) in the Polish version which is tailored to evaluate various cognitive domains such as attention and concentration, executive functions, memory, language, visuo-constructional skills, conceptual thinking, calculations, and orientation. The total possible score was 30 points with 26 points or above considered as cognitive health and scores below 26 points as potential mild cognitive impairment (MCI). The duration of the test was about 10 min. All cognitive tests were conducted by experienced psychologists.

### Brain MRI Acquisition and Analysis

Brain MRI was performed using the same 1.5 Tesla MR scanner (GE, Hdx). The scanning protocol included: 3D T1-weighted images [repetition time (TR) = 8.3 ms, echo time (TE) = 3.2 ms, inversion time (TI) = 650 ms, field of view (FOV) = 240 × 240 mm^2^, slice thickness =1 mm, flip angle = 12°, matrix = 256 x 256, NEX = 1), T2-weighted images (TR = 2720 ms, TE = 88/8.8ms, FOV = 240 × 240 mm^2^, slice thickness = 3.5 mm, matrix = 256 x 256, NEX = 1), Fluid-attenuated inversion recovery sequences (FLAIR) (TR = 8.800 ms, TE = 145 ms, TI = 2.2 ms, FOV = 240 × 240 mm^2^, slice thickness = 3.5 mm, matrix = 256 x 256, NEX = 1), diffusion weighted images (DWI) (TR = 1000 ms, echo TE = 107 ms, FOV = 240 x 168 mm^2^, slice thickness = 0.7 mm, flip angle = 20°, matrix = 256 x 256, NEX = 1), susceptibility-weighted images (SWI) (TR = 73.9 ms, TE = 47.4 ms, FOV= 240 x 168 mm^2^, slice thickness = 3.5 mm, flip angle = 12°, matrix = 256 × 256, NEX = 1).

### Visual Assessment of MR Images

Vascular lesions related to CSVD were classified according to the standard published criteria as lacunar infarcts, microbleeds and WMH (20). Lacunar infarcts were assessed on T2-weighted and FLAIR images as infarcts in basal ganglia, thalami and the white matter smaller than 1.5 cm in size. Presence of microbleeds was assessed on SWI images. White matter hyperintensities were assessed on T2-weighted and FLAIR images separately in the periventricular (periventricular WMH) and subcortical (subcortical WMH) locations and graded based on the Fazekas scale (10) with grades 0 or 1 interpreted as normal and grades 2 or 3 regarded as pathological amount of WMH.

All MR scans were rated by two independent experienced neuroradiologists trained for CSVD assessment using the same criteria. The inter-rater reliability was established with kappa coefficient that ranged between 0.71 and 0.88 depending on the category of the assessed parameters. Despite the good inter-rater reliability, all scans that showed any discrepancy between the two readers were re-evaluated and the final score was established by consensus.

### Volumetric Measurements of the MRI Data

Data processing workflow consisted of a voxel-based processing using the Computational Anatomy Toolbox 12 (CAT12, Structural Brain Imaging Group, University of Jena) and the Statistical Parametric Maps 12 (SPM12) software. The initial voxel processing steps included a spatial adaptive non-local means (SANLM) denoising filter (21) then data was bias-corrected and affine-registered followed by the standard SPM “unified segmentation” (22). Further steps included skull-stripping of the brain, parcellation into the left and right hemisphere, subcortical areas, and the cerebellum. To address problems of gray matter intensities a local intensity transformation of all tissue classes was performed. Final steps included adaptive maximum a posteriori (AMAP) segmentation (23), which was then refined by applying a partial volume estimation (24). We have used a CAT12 method to detect and handle WMH based on the 3D T1-weighted sequence which is comparable to existing T2/FLAIR-based methods (25) and uses the region-growing approach to identify all WMH lesions also those in the periventricular location (26).

**Figure 1:**
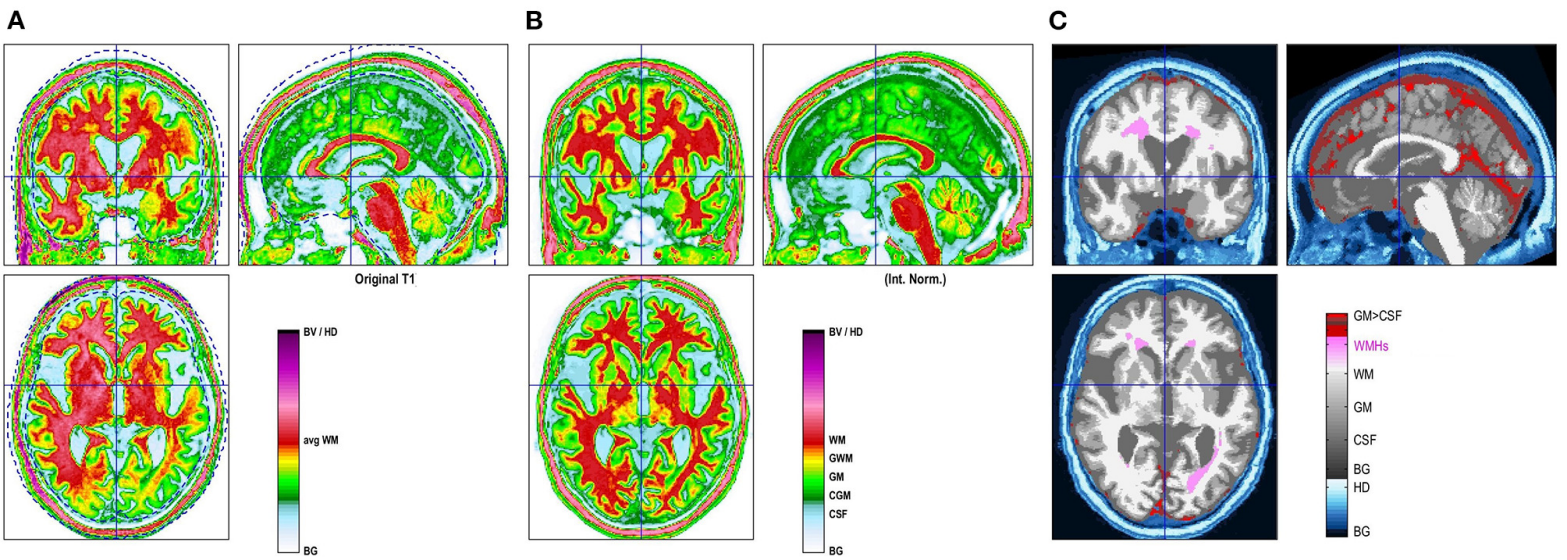
CAT12 VBM images showing segmented brain tissues: original image **(A)**, normalized image with template **(B)** and WMH segmentation **(C)**.

For quality assurance, a 2-step process was used. First, before preprocessing in CAT12, overall data quality was checked and datasets with artifacts were rejected. Secondly, the quality control measures incorporated in the CAT12 processing pipeline were used to identify the corrupted data after segmentation. Additionally, all segmentations for each subject were manually reviewed for its quality. As a results 23 subjects were rejected due to incomplete or insufficient data quality due to movement artifacts or aborted scanning. Finally 554 subjects were included in the study. Tissue class images in spatial correspondence to the original data have been obtained. This allowed us to calculate native volumes of the gray and white matters, cerebrospinal fluid and WMH for each subject which were exported from CAT12 for statistical analyses. Figure 1 shows the segmented brain tissue images generated using the CAT12 VBM methods.

Final volumetric measurements consisted of the volumes of the total intracranial space, total gray matter (GMvol), total white matter (WMvol), total cerebrospinal fluid and WMH (WMHvol). Volumes of total gray matter, total white matter and total WMH were estimated as direct volumes in mm^3^ as well as the volumes normalized to the volume of the total intracranial space to reduce the influence of individual and sex-related differences in brain sizes.

### Statistical Analysis

Descriptive statistics of the demographic data, risk factors and the results of brain volumetry were presented as mean values with standard deviations and range, or number of cases with percentage.

Analysis of the effect of different risk factors on GMvol, WMvol and WMHvol was performed using multiple linear regression modeling. Statistics presented for those models are regression coefficient and its 95% confidence interval.

Analysis of the influence of different risk factors on pathological MOCA scores (0–25 vs. 26–30 points) as well as pathological accumulation of periventricular and subcortical WMH (Fazekas grades 2–3 vs. Fazekas grades 0–1) was assessed using a binary logistic regression modeling. Statistics presented for those models are odds ratio and its 95% confidence interval.

In each case full models were used adjusted for age, sex and all risk factors. Models for GMvol, WMvol and MOCA score were additionally adjusted for WMHvol. *P*-values in multivariate regression analyses were corrected for multiple testing using Benjamini-Hochberg correction.

All calculations were made using the R package for Windows (version 4.0.4). Statistical results were considered significant when the *p*-value was < 0.05.

## Results

**Figure 2:**
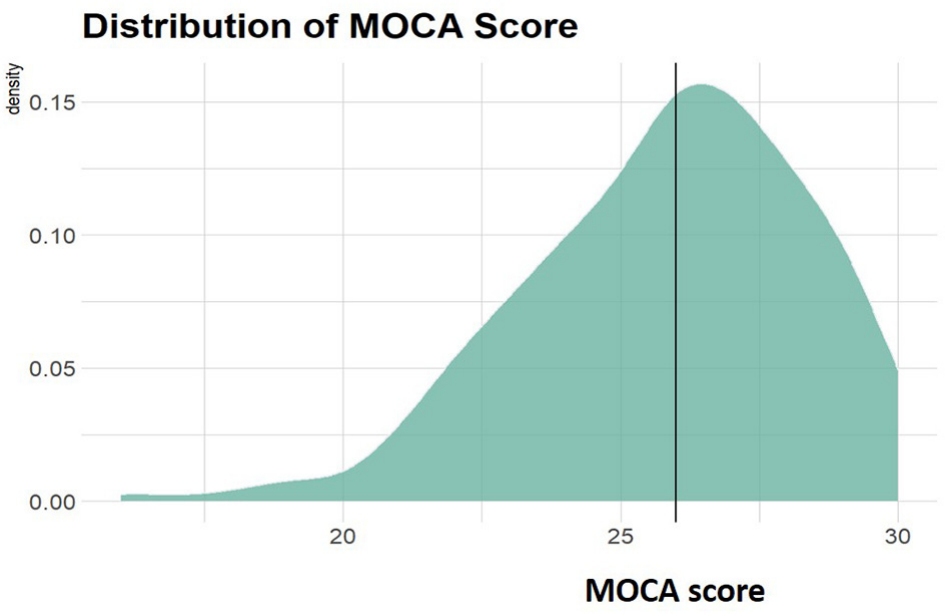
Distribution of MOCA scores among participants. Vertical line indicates 26 points which is a threshold between normal (26–30) and abnormal (0–25) scores.

The largest group of participants consisted of the older population between 60 and 69 years of age (335/554, 60.47%), followed by the group between 50 and 59 years (219/554, 39.43%) (Table 1). Women constituted the majority of the study group (367/554, 66.25%). Hyperlipidemia occurred in about 59.57% of the subjects, followed by hypertension in ~50% of the group, while diabetes was found in 18.6 % of the subjects. Majority of the subjects (70.76%) had normal body weight (BMI <25), 81.41% never smoked and around 24.19% declared no alcohol consumption. Approximately 40% of the participants scored <26 points in the MoCA test (Table 1, Figure 2).

**Table 1:** Characteristics of the study group.

**Factors**	**Characteristics**	**Number (%) Mean value ± SD**
Age	Mean age	61.49 ± 5.6
	50–59 yrs	219 (39.43%)
	60–69 yrs	335 (60.47%)
Sex	Male	187 (33.75%)
	Female	367 (66.25%)
Diabetes	Yes	103 (18.59%)
	No	451 (81.41%)
Hypertension	Yes	276 (49.82%)
	No	278 (50.18%)
Hyperlipidemia	Yes	330 (59.57%)
	No	224 (40.43%)
Body Mass Index	Normal <25	392 (70.76%)
	Abnormal ≥ 25	162 (29.24%)
Smoking	Yes	103 (18.59%)
	No	451 (81.41%)
Alcohol consumption	Yes	420 (75.81%)
	No	134 (24.19%)
The Montreal Cognitive Assessment (MoCA) score	Normal ≥ 26 points	332 (59.93%)
	Abnormal <26 points	222 (40.07%)
	Mean score	25.77 +/−2.775
	[range]	[16–30]

**Figure 3:**
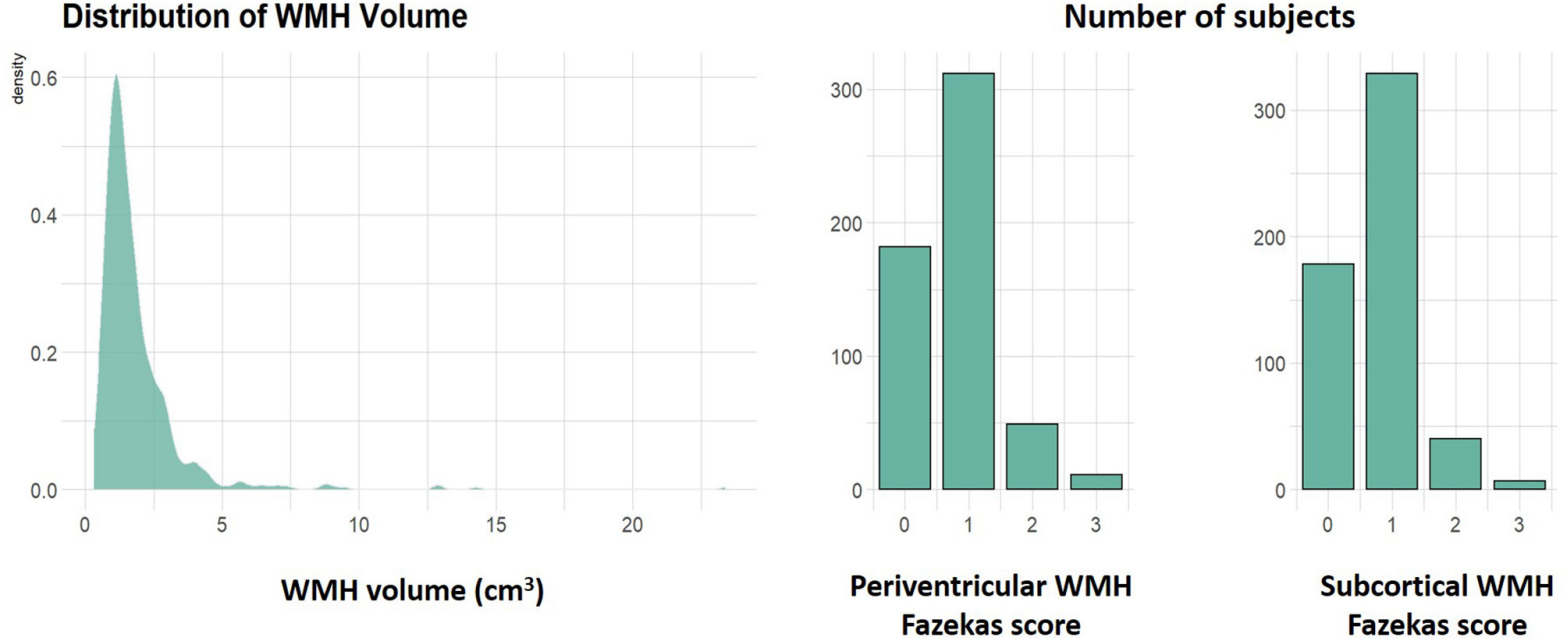
Distribution of white matter hyperintensities (WMH) volumes among participants as well as histograms showing numbers of subjects with normal (scores 0–1) and pathological (scores 2–3) amount of periventricular and subcortical WMH according to the Fazekas scale.

The results of both visual inspection as well as volumetric analysis of MR images are shown in Table 2. Visual inspection of MR images revealed lacunar infarcts and microbleeds in <30 subjects each. Pathological amount of WMH (Fazekas grades two or three either in the periventricular or subcortical location) was present in 13.36% of the study group, more often in the periventricular (10.83%) than in the subcortical (8.48%) location. Distribution of WMH burden according to Fazekas scale is shown in Figure 3. Parametric results of GMvol, WMvol and WMHvol both direct in cm^3^ and normalized to the volume of the total intracranial space are shown in Table 2.

**Table 2:** Volumetric measurements of the gray and white matters as well as cerebral small vessel disease characteristics derived from brain MRI.

**Parameter**	**Characteristics**	**Parametric value**
Gray Matter	Volume (cm^3^)	Mean +/–SD 571.3 +/−49.95
	Normalized volume	Mean +/–SD 0.396 +/−0.0226
White Matter	Volume (cm^3^)	Mean +/–SD 501.1 +/−58.75
	Normalized volume	Mean +/–SD 0.3436 +/−0.0216
White Matter Hyperintensities (WMH)	Volume (cm^3^) in all subjects	Mean +/–SD 1.85 +/−1.73
		Median 1.4
		Min 0.31
		Q1 1.01
		Q3 2.11
		Max 23.37
	Normalized volume in all subjects	Mean +/–SD 0.0013 +/−0.0012
		Median 0.001
		Min 0.0003
		Q1 0.0007
		Q3 0.0014
		Max 0.014
	Volume (cm^3^) in all subjects with pathological WMH (Fazekas grade 2 or 3 either in the periventricular or subcortical location)	Number (%) 74 (13.36)
		Mean +/- SD 4.14 +/- 3.52
		Median 2.93
		Min 1.08
		Q1 2.16
		Q3 4.34
		Max 23.37
	Periventricular (Fazekas grade 2 or 3)	Number (%) 60 (10.83)
	Subcortical (Fazekas grade 2 or 3)	Number (%) 47 (8.48)
Lacunar infarcts	Yes	Number (%) 28 (5.05)
Microbleeds	Yes	Number (%) 22 (3.97)

The results of the multivariate regression models used for the evaluation of associations between age, sex and multiple risk factors and brain parameters derived from MRI as well as the results of the MoCA test are shown in Tables 3, 4. Statistical analysis revealed that older age was significantly associated with lower GMvol (*p* < 0.001, corrected *p* < 0.001), lower WMvol (*p* < 0.001, corrected *p* < 0.001) and higher WMHvol (*p* < 0.001, corrected *p* = 0.0003). Age was a significant factor increasing the risk of pathological amount of periventricular (*p* < 0.001, corrected *p* = 0.0001) and subcortical (*p* = 0.005, corrected *p* = 0.01) WMH, as well as lower scores at MoCA test (*p* < 0.001, corrected *p* = 0.005). Even though all volumetric measurements were corrected for the volume of the intracranial space to avoid significant individual or sex-related differences in brain sizes, still male sex was associated with the higher GMvol compared to females (*p* < 0.001, corrected *p* < 0.001).

**Table 3:** Associations between multiple risk factors and the volumes of the gray and white matter as well as MoCA scores.

**Risk factors**	**Total gray matter volume (normalized)**			**Total white matter volume (normalized)**			**MoCA score**		
	**Regression coefficient**	**“95% Cl”**	**p (corrected p)**	**Regression coefficient**	**“95% Cl”**	**p (corrected p)**	**Odds ratios**	**“95% Cl”**	**p (corrected p)**
Age	−0.0015	−0.0018 to −0.0012	**<** **0.001 (< ** **0.001)**	−0.0016	−0.0019 to −0.0012	**<** **0.001 (< ** **0.001)**	1.0857	1.0453 to 1.1291	**<** **0.001 (0.005)**
Sex (Male)	−0.0155	−0.0189 to −0.0122	**<** **0.001 (< ** **0.001)**	−0.0033	−0.0070 to 0.0003	0.07 (0.21)	1.1918	0.8088 to 1.7546	0.37 (0.61)
Hypertension	−0.0007	−0.0042 to 0.0027	0.68 (0.78)	−0.0006	−0.0043 to 0.0032	0.76 (0.83)	0.8735	0.5863 to 1.2963	0.5 (0.61)
Diabetes	0.0018	−0.0023 to 0.0059	0.39 (0.60)	0.0009	−0.0036 to 0.0054	0.69 (0.83)	1.2956	0.8111 to 2.0677	0.27 (0.53)
Hyperlipidemia	0.0004	−0.0029 to 0.0037	0.81 (0.86)	−0.0008	−0.0043 to 0.0028	0.67 (0.83)	0.8869	0.6110 to 1.2903	0.52 (0.61)
Smoking	−0.0026	−0.0066 to 0.0015	0.21 (0.35)	−0.0015	−0.0058 to 0.0029	0.51 (0.83)	1.1790	0.7363 to 1.8776	0.48 (0.61)
Alcohol	−0.0028	−0.0064 to 0.0009	0.13 (0.28)	−0.0018	−0.0058 to 0.0021	0.36 (0.73)	0.6906	0.3894 to 0.8943	0.06 (0.05)
BMI	0.0008	−0.0029 to 0.0045	0.68 (0.80)	−0.0035	−0.0076 to 0.0005	0.08 (0.21)	0.6614	0.4233 to 1.0256	0.06 (0.17)
WMH volume	−0.0031	−0.0040 to −0.0022	** <0.001 (< ** **0.001)**	0.0001	−0.0009 to 0.0011	0.82 (0.83)	1.0498	0.9431 to 1.1796	0.38 (0.61)

*Analysis of the effect of different risk factors on GMvol and WMvol was performed using multiple linear regression modeling [statistics presented for those models are regression coefficient and its 95% confidence interval (CI)] while the effect on pathological MOCA scores was assessed using a binary (0–25 points vs. 26–30 points) logistic regression modeling (statistics presented for this model are odds ratio and its 95% CI). Corrected p indicates p-values after the Benjamini-Hochberg correction. Bold values indicate statistically significant*.

**Table 4:** Associations between multiple risk factors and white matter hyperintensities (WMH).

**Risk factors**	**Total WMH volume (normalized)**			**Periventricular WMH**			**Subcortical WMH**		
	**Regression coefficient**	**“95% Cl”**	**p (corrected p)**	**Odd ratios**	**“95% Cl”**	**p (corrected p)**	**Odd ratios**	**“95% Cl”**	**p (corrected p)**
Age	0.0039	0.0020 to 0.0057	** <0.001 (0.0003)**	1.1561	1.0842 to 1.2385	** <0.001 (0.0001)**	1.0979	1.0296 to 1.1751	**0.005 (0.01)**
Sex (Male)	0.0117	−0.0089 to 0.0323	0.26 (0.41)	0.6887	0.1811 to 0.771	0.2 (0.51)	0.6763	0.3278 to 1.3222	0.26 (0.58)
Hypertension	0.0256	0.0045 to 0.0468	**0.01** (0.06)	2.5573	1.3122 to 5.2137	**0.007 (0.02)**	2.9605	1.4316 to 6.5103	**0.005 (0.01)**
Diabetes	0.0053	−0.0202 to 0.0308	0.68 (0.78)	0.8321	0.3839 to 1.7003	0.62 (0.79)	0.9971	0.4484 to 2.0848	0.99 (0.99)
Hyperlipidemia	0.0217	0.0017 to 0.0417	**0.03** (0.08)	0.9858	0.5397 to 1.8195	0.96 (0.94)	1.2467	0.6565 to 2.4178	0.5 (0.58)
Smoking	0.0158	−0.0091 to 0.0406	0.21 (0.39)	1.5396	0.6987 to 3.1855	0.26 (0.47)	1.3034	0.5562 to 2.7950	0.51 (0.58)
Alcohol	−0.0023	−0.0246 to 0.0200	0.83 (0.85)	0.9149	0.4811 to 1.8168	0.79 (0.90)	1.3358	0.6536 to 2.9682	0.44 (0.58)
BMI	−0.0057	−0.0286 to 0.0172	0.62 (0.78)	1.2406	0.5737 to 2.5928	0.57 (0.79)	1.3232	0.5783 to 2.8984	0.49 (0.58)

*Analysis of the effect of different risk factors on WMHvol was performed using multiple linear regression modeling [statistics presented for this model are regression coefficient and its 95% confidence interval (CI)] while the effect on pathological accumulation of Periventricular and Subcortical WMH was assessed using a binary (Fazekas grades 2–3 vs. Fazekas grades 0–1) logistic regression modeling (statistics presented for those models are odds ratio and its 95% CI). Corrected p indicates p-values after the Benjamini-Hochberg correction. Bold values indicate statistically significant*.

Evaluation of the impact of multiple risk factors revealed that hypertension had a significant influence on the presence of a pathological amount of WMH in both periventricular and subcortical locations before and after correction for multiple comparisons (before correction *p* = 0.007 and *p* = 0.005, respectively, and after correction *p* = 0.02 and *p* = 0.01, respectively). Hypertension and hyperlipidemia showed significant impact on WMHvol before correction (*p* = 0.01 and *p* = 0.03, respectively), but not after correction (*p* = 0.06 and *p* = 0.08, respectively).

On the other hand, risk factors such as diabetes, smoking, alcohol consumption or abnormal BMI showed no significant influence (*p* > 0.05) on GMvol, WMvol or the presence of WMH before and after correction for multiple comparisons.

The presence of higher WMHvol was significantly associated with lower GMvol before and after correction (*p* <0.001), without any effect on WMvol or the MOCA score (*p* > 0.05).

The MoCA score was not associated with GMvol, WMvol and WMHvol or with any other risk factor, except for age (*p* > 0.05), both before and after correction for multiple comparisons.

## Discussion

The main goal of this study was to assess the impact of age, sex and multiple risk factors on aging and degeneration of the gray and white matter characterized by the decrease of their volumes and the presence of a pathological burden of WMH reflecting chronic white matter damage.

Even though the age range in the studied population was only 20 years and all the subjects were between 50 and 70 years of age, still age was found to be one of the most important factors influencing the volumes of the gray and white matter and WMH as well as the presence of a pathological burden of periventricular and subcortical WMH. It is not surprising since all these features are hallmark of both physiological and pathological aging of the brain. Our results are in accordance with many previous histological and neuroimaging reports that established normal aging to be associated with a distinctive atrophy of mainly hemispheric gray and white matter with a reduction of brain volumes at a rate of 5% per decade after 40 years of age, together with the increased accumulation of WMH (2, 4, 10, 20, 27–32).

Sex-related differences in the size of male and female brains have already been reported (33, 34). Both, to account for normal variation in head size and sex-related smaller volumes of the brain in women, all volumetric measurements were normalized to the total volume of the intracranial space. We did not find any influence of male or female sexes on the volumes of the white matter and WMH, though the gray matter volume was significantly increased in men compared to women.

Analysis of the impact of multiple factors on the volume of the gray matter revealed that only higher WMH volume was associated with lower volumes of GMvol. No influence of any other of the evaluated risk factors on GMvol was demonstrated. There are numerous reports in the literature about the indisputable effect of WMH on brain atrophy and gray matter loss (12, 13, 15, 19, 35–38). Moreover, Habes et al. found that individuals with a high burden of WMH displayed significantly higher rate of the cortical atrophy pattern similar to that found in AD, compared to people with a lower burden of WMH exhibiting more age-related patterns of brain atrophy (14). Several mechanisms have been proposed to explain the association between WMH and gray matter atrophy, such as ischemic damage or Wallerian degeneration. It is believed that there are several mechanisms for the formation of WMH, and the most important is the direct injury of the myelin sheaths of axons, oligodendrocytes, glial cells as a result of small vessel damage. Ischemia of the subcortical axons leads to the disappearance of gray matter cells through damage to synapses. As a result of microangiopathy, cerebral circulation is dysregulated leading to hypoxia and secondarily to loss of neurons (35, 36). Advanced neuroimaging methods suggest that WMH particularly affect white matter networks connecting remote brain regions and thus lead to gray matter shrinkage through Wallerian degeneration (39). These results make it particularly important to assess an individual's brain health profile with both the presence of WMH and gray matter loss that is influenced by the coexistence of WMH. Thus, a well-known relation between WMH and cognitive decline or even dementia should be explained by two mechanisms such as direct damage of the white matter and acceleration of the gray matter loss.

In our study evaluation of the white matter consisted of the assessment of its volume and the presence of WMH. Age was found to be the only factor with an impact on the WMvol. On the other hand, hypertension was demonstrated to significantly increase the volume of WMH and the risk of pathological amount of WMH located both in the periventricular and subcortical brain regions. Moreover, hyperlipidemia was found to be associated with the higher volumes of WMH though this relationship was not as strong as with hypertension and only significant before correction for multiple comparisons. Nevertheless, our findings are in accordance with several previous reports from the Western European, North American and Chinese studies (15, 18). Hypertension is one of the most commonly identified factors associated with WMH (14). Several mechanisms, including neuroinflammation, oxidative stress, dendritic shrinkage, and apoptosis, are thought to be implicated in the pathophysiology linking elevated blood pressure and neurodegeneration. It has been postulated that hypertension may increase accumulation of WMH by disrupting the blood-brain barrier integrity what causes a secondary disorder of neuronal nutrition that results in their apoptosis. More often also cardio-vascular factors are thought to be very important contributors. Since high blood pressure increases the risk of arthrosclerosis by 50% or more, it is likely to lead to lower blood perfusion in capillaries, endothelia dysfunction, impaired vasoreactivity, increased pulsatility, vessel stiffening, and changes to the blood brain barrier integrity. Small vessel disease in conjunction with ischemia, inflammation, and myelin loss are then likely to contribute to the development of WMH (14, 39).

Observations of the impact of hyperlipidemia on the human body are numerous, while the literature has no clear data regarding its direct relationship with the process of brain damage. The causes may be related to the accumulation of cholesterol molecules in the inner membrane of the arteries, resulting in damage to the vascular endothelium. It might also lead to a decrease in the level of secreted prostacyclins, which prevent platelets from sticking, therefore clots and stenosis may form, and vessel walls may harden. The above pathogenesis applies to all arterial vessels, including cerebral vessels. These processes lead to secondary ischemia of the nerve cells and their atrophy (40). It has to be stressed that the etiology of WMH is of particular significance, as they impact cognitive function across all domains, probably in a greater extent than brain atrophy (39).

In our study, factors without any influence on the gray or white matter volumes or WMH were diabetes, abnormal BMI, smoking and alcohol intake. Regarding diabetes there is no clear scientific evidence for a significant effect of glycemic disorders on the process of brain atrophy and there are contradictory reports on its influence on brain structures (14, 15, 41–44). The causes of brain damage and accelerated process of volume reduction and WMH accumulation in diabetics may be explained by direct toxic effects of the elevated glucose levels on nerve cells, damage of cerebral vessels, as well as by the disturbance of biochemical processes, among others inappropriate stimulation of receptors by insulin. Increased BMI has already been reported to be associated with a decrease in the total brain volume, though it was not demonstrated in our study (15, 45). The available literature lacks detailed analysis of the impact of abnormal BMI on the atrophy of individual brain structures. The mechanism by which obesity reduces the volume of the brain is not fully understood. Possible mechanisms include the metabolic syndrome that coexists with obesity and factors such as high triglyceride levels, low HDL cholesterol, hypertension, insulin resistance, as well as thrombotic and inflammatory condition that may reduce cerebral perfusion and increase β-amyloid production leading to neuronal degeneration. Leptin, an obesity-related peptide, has also been reported to increases the deposition of β-amyloid (45).

In this study, factors such as smoking or alcohol consumption had no significant impact on the volumes of the evaluated brain regions. It may be related to a relatively small number of active smokers (18%) and only occasional alcohol drinkers in the study material. However, it has to be stressed that there are reports in the literature that showed the effect of a long-term cigarette smoking on generalized brain atrophy and WMH accumulation either through the direct cytotoxic effect of tar substances, e.g., carbon monoxide (CO) on nerve and glial cells leading to their apoptosis through damage to cell membranes or the indirect mechanism associated with lowering the concentration of natural antioxidants, including vitamins A, C, E, which makes nerve cells more prone to oxidative stress and injury (14, 15, 46). Smoking is also associated with a more intense occurrence of atherosclerosis, which leads to secondary ischemia of nerve cells and their death (46).

On the other hand, studies on alcohol consumption showed that it is another important epidemiological factor related with brain atrophy, especially cerebellum (47–49). It has been reported that there is a significant acceleration of physiological brain atrophy in the group of people who consume a large amount of alcohol, ~418.1 g per week. However, moderate or low alcohol consumption below 181.2 g per week has no significant effect on brain volume (50). What is interesting the effect of alcohol consumption on total brain atrophy is represented by the so-called J or U curve since both groups of people non-drinkers and severe abusers show a tendency to higher brain atrophy which increases the risk of cognitive impairment (MCI) and dementia, while low doses of alcohol do not have an effect on brain volume or may even be protective (47–50). In our study no significant impact of the alcohol consumption on the brain volume may be associated with a moderate alcohol intake by the participants which is believed to have the weakest influence on brain atrophy.

In our study the increased risk of cognitive deficits in the MoCA score was only associated with the increased age. The MoCA score was not influenced by any of the evaluated risk factors or the volumes of the total gray and white matter as well as WMH. The MoCA is a brief global cognitive assessment which is sensitive to vascular cognitive impairment (51). The reports on the associations of MoCA and WMH are contradictory. Similarly to us, Smith at al did not report a significant impact of total gray and white matter volumes as well as WMH volume on MoCA in the Canadian cohort concluding that this test may not be sensitive enough to the presence of covert cerebrovascular disease in a population-based setting (19). On the other hand, recent larger Canadian study by Anand et al. demonstrated such associations between MoCA and WMH. Moreover, there have been several reports in the literature revealing significant impact of obesity or hypertension on worse scoring in MoCA as well as a protecting role of not smoking (52–54). It should be stressed that subjects in our study had relatively high scores of MoCA with mean 25.77 (below 26 is regarded as cognitive impairment) and did not report any subjective complaints on cognitive dysfunction. We believe that the larger cohort of participants or an inclusion of subjects with more pronounced cognitive impairment with lower MoCA scores could influence the results of associations between MoCA and WMH.

Major strength of our study is that this is the first large scale study of this kind in Central Europe based on the big cohort of participants recruited in the Polish fraction of the multinational PURE study focused on the evaluation of the socio-demographic and health-related factors and their impact on general health, brain lesions and cognition. Another strength is the parallel assessment of the influence of multiple factors and their association with brain volumes, WMH and the results of a psychological test. All participants underwent exactly the same procedures regarding their physical and psychological health assessment and were scanned on the same MR scanner using the same protocol what provided good quality reproducible data sets.

There are several limitations of the study. First, the cross-sectional observational character of the study and assessment of the volumes of total gray and white matter without detailed evaluation of multiple separate structures of the brain such as basal ganglia or cerebellum. On the other hand what is known from the literature brainstem and basal ganglia seem to be less affected regarding volumetric loss. In those structures changes due to aging are found mostly in their microstructure and thus may be visualized using other more sensitive imaging techniques such as quantitative MRI (qMRI) or tensor-based morphometry (TBM) (55). Another limitation is the use of the 1.5 Tesla MR scanner that allows for reliable assessment of volumetric measurements of the gray and white matters as well as WMH, but does not allow for the application of more advanced techniques such as diffusion tensor imaging or rs-fMRI which may give more detailed insight into subtle microstructural and functional abnormalities due to aging. A further limitation of the study is the use of 3D T1 sequences to assess WMH volume which is not a standardized and generally accepted method, although it was already used by Dahnke et al. (25) on a small sample group and by Dadar et al. (56) who validated this method on large-scale datasets. According to Dahnke et al. this method has a risk of underestimation of WMH volume when the lesion volume is under 10 cm^3^. On the other hand, Dadar et al. proved that regarding the assessment of clinical outcomes, the WMH segmentation based only on T1-weighted imaging correlates with age, cognitive, and clinical measures as strongly as the WMH volumes determined using the optimal modalities of FLAIR or T2w/PD.

## Conclusions

Based on the results of our study apart from age, the main risk factor of the gray matter volume reduction is the presence of WMH. On the other hand degeneration of the white matter reflected by the increased accumulation of WMH is strongly related to hypertension and also to hyperlipidemia. Thus, prevention of gray and white matter damage should be focused especially on potentially modifiable risk factors affecting WMH accumulation of which hypertension and hyperlipidemia seem to be of the greatest importance. Our results from the largest population-based cohort study from Central Europe follow the results of the analogous studies from North America, Western Europe and China. We believe that prevention of pathological accumulation of WMH and gray matter loss may be an important factor reducing signs of pathological aging of the brain which when uncontrolled may lead to cognitive impairment or even dementia.

## Data Availability Statement

The original contributions presented in the study are included in the article/supplementary material, further inquiries can be directed to the corresponding authors.

## Ethics Statement

The studies involving human participants were reviewed and approved by the local University Ethics Committee for conducting research involving humans. Each participant signed his/her informed consent before inclusion. The patients/participants provided their written informed consent to participate in this study.

## Author Contributions

JJ-N conducted all volumetric measurements, analyzed and interpreted data, conducted literature search, and wrote the manuscript. PP contributed to the study design and supervised all volumetric measurements, and prepared all figures. JB contributed to the study design and critically reviewed the manuscript. DS performed psychological assessment of all subjects and critically reviewed the paper. JR performed psychiatric assessment of the subject and critically reviewed the paper. KZ contributed to the study design, provided all epidemiological data, and critically reviewed the paper. KP-Z contributed to the study design, provided epidemiological data, and critically reviewed the paper. MS contributed to the study design and critically reviewed the paper. AS contributed to the study design, critically reviewed the paper, and supervised the research project. AZ contributed to the study design, critically reviewed the paper, supervised the research project, the study design, analyzed and interpreted MRI data, supervised writing of the manuscript, and critically reviewed the paper. All authors contributed to the article and approved the submitted version.

## Conflict of Interest

The authors declare that the research was conducted in the absence of any commercial or financial relationships that could be construed as a potential conflict of interest.
